# Evaluation insight into Abu Zenima clay deposits as a prospective raw material source for ceramics industry: Remote Sensing and Characterization

**DOI:** 10.1038/s41598-022-26484-5

**Published:** 2023-01-02

**Authors:** Ali Maged, Sherif Ahmed Abu El-Magd, Ahmed E. Radwan, Sherif Kharbish, Sara Zamzam

**Affiliations:** 1 Geology Department, Faculty of Science, Suez University, P.O. Box 43518, El Salam City, Suez Governorate Egypt; 2 Faculty of Geography and Geology, Institute of Geological Sciences, Jagiellonian University, Gronostajowa 3a, 30-387 Kraków, Poland; 3 Geology Department, Faculty of Science, Zagazig University, Zagazig City, 44519 Sharkia Governorate Egypt

**Keywords:** Geochemistry, Planetary science, Solid Earth sciences, Geochemistry, Geology, Mineralogy, Sedimentology

## Abstract

The rapid development and mutations have heightened ceramic industrialization to supply the countries' requirements worldwide. Therefore, the continuous exploration for new reserves of possible ceramic-raw materials is needed to overwhelm the increased demand for ceramic industries. In this study, the suitability assessment of potential applications for Upper Cretaceous (Santonian) clay deposits at Abu Zenima area, as raw materials in ceramic industries, was extensively performed. Remote sensing data were employed to map the Kaolinite-bearing formation as well as determine the additional occurrences of clay reserves in the studied area. In this context, ten representative clayey materials from the Matulla Formation were sampled and examined for their mineralogical, geochemical, morphological, physical, thermal, and plasticity characteristics. The mineralogical and chemical compositions of starting clay materials were examined. The physicochemical surface properties of the studied clay were studied utilizing SEM–EDX and TEM. The particle-size analysis confirmed the adequate characteristics of samples for white ceramic stoneware and ceramic tiles manufacturing. The technological and suitability properties of investigated clay deposits proved the industrial appropriateness of Abu Zenima clay as a potential ceramic raw material for various ceramic products. The existence of high kaolin reserves in the studied area with reasonable quality and quantity has regional significance. It would significantly help reduce the manufacturing cost and overwhelm the high consumption rate. The ceramic manufacturers in the investigated areas are expected to bring steady producers into the industry in the long term to gain the advantage of low-cost raw materials, labor, and factory construction.

## Introduction

Natural clays are generally abundant and well-known ancient materials utilized by humans and are still widely used in various applications. Clays, in particular, have an outstanding utilization record in several industries, such as building bricks, infrastructure, insecticide, water filters, rubber, pharmaceutical, and personal care products. Furthermore, clays have recently been involved in the design of new materials and composites for various development purposes, such as traditional and engineered ceramics^1^, lightweight aggregates^2^, hybrid metalo-ceramic composites^3^, geopolymers^4^, adsorbent^5^, lightweight syntactic foam composite^6^, lipid preservation^7^, cementitious construction materials^8^, low-cost ceramic membrane^9^, and pharmaceutical carriers in drug delivery systems^10^.

Clays are naturally composed of clay minerals, non-clay sediments, and a particular amount of moisture. Clays are usually a combination of layered-structure hydrated aluminosilicates existing with ratios of silicon dioxide (SiO_2_)/aluminum oxide (Al_2_O_3_) ranging from 2.0/1.0 to (4.0–5.0)/1.0^1^. Clays can be subdivided into several categories, including montmorillonite-, kaolinite-, illite-, and chlorite-rich clays. The selection of suitable clays as a primary raw material for clay-based ceramic products can be determined based on their mineralogical, chemical, physical, and particle size properties^11^. Globally, kaolinite-rich clays are the most plentiful and extensively employed aluminosilicate minerals, especially in Egypt^12^. Kaolinite (${\mathrm{Al}}_{2}{\mathrm{O}}_{3}\cdot 2{\mathrm{SiO}}_{2}\cdot 2{\mathrm{H}}_{2}\mathrm{O}$) is considered as the abundant minerals that exist in the Earth's crust. Consequently, kaolin and kaolinite-rich clays are broadly utilized in the manufacturing of traditional ceramics. Historically, kaolin deposits were originally the first raw material used in the ceramic industry and are still the best-known industrial application of kaolin deposits. The most important properties naturally existing in kaolin for ceramic purposes are plasticity, particle size, and fired color. Moreover, there are various types of clays which used in the ceramic industry such as white-firing plastic clays (ball clays)^13^, medium–low plasticity white-firing clays^14^, red-firing plastic clays^15^, red-firing clays with carbonates^16^. Ceramic clays could be in the original depositional conditions or have been subjected to burial, with the related effects of diagenesis. These conditions could lead to the progressive consolidation of the sediment (i.e., from clay to claystone to shale) up to lithification (slate) with consequent modifications of the mineralogical composition (i.e., illite–smectite interstratified > illite > sericite) and physical properties (especially plasticity and grindability). Ceramic plants can process from unconsolidated sediments to moderately consolidated clay materials, but strongly lithified claystone usually needs to be treated as "hard materials" (i.e., feldspathic rocks)^16,17^.

The rising population is one of the major factors driving the growth in demand for the ceramic market. Furthermore, the population shift from the countryside to urban areas has dramatically increased. This shift in living standards will require enhanced sanitation and hygiene. Additionally, COVID-19 has severely impacted the construction industry due to lockdowns. Consequently, there is an ever-increasing market demand for kaolin minerals, construction materials, and ceramic products. Therefore, the continuous exploration for new reserves of kaolin deposits is needed to overwhelm the increased demand for ceramic industries and the resource shortage.

The present study aimed to evaluate the unexploited kaolinite-rich clays from the Abu Zenima area, South Sinai, Egypt, for various ceramic applications. First, remote sensing techniques were used to assess the kaolin reserves in the studied area for the regional delineation of Kaolinite-bearing formations. Thereafter, the geological setting and lithological units of Abu Zenima area were intensively discussed. Furthermore, the experimental design undertakes as follows: (1) pinpoint the main characteristics of the collected clay samples in terms of physical, chemical, thermal, phases, and microstructural properties, including X-ray fluorescence spectroscopy (XRF), X-ray diffraction (XRD), Cation Exchange Capacity (CEC), Fourier transform infrared (FT-IR) analysis, Scanning Electron Microscopy (SEM), Transmission Electron Microscope (TEM), Energy Dispersive X-ray spectrometer (EDX), nitrogen adsorption/desorption measurements, and thermal gravimetric (TG) and differential thermal (DTA) analysis; (2) the grain size analysis using two different methods was also investigated; (3) the plasticity, Bigot curves, and granulometric study were precisely applied in order to get insight into the appropriateness of the examined clay samples for the manufacturing of ceramic products; and (4) the regional significance of the present study was presented. Consequently, this study can successfully fill the gap in the studies of clay deposits of the Middle east region to help the local economy by attracting the consuming industries.

## Methods

### Study area description

**Figure 1:**
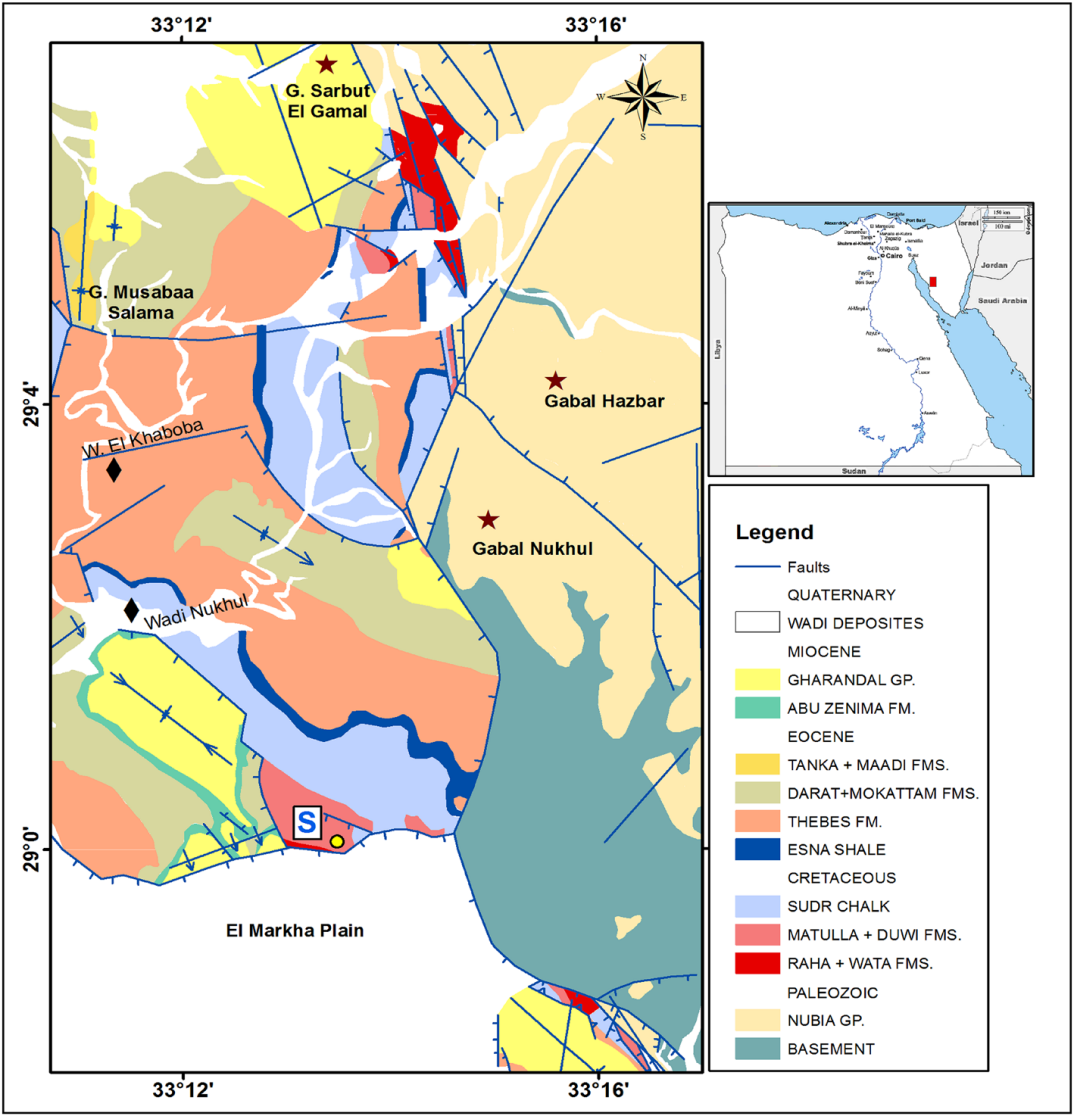
Geological map of the study area occurred along the Eastern side of the Gulf of Suez (modified after Moustafa (1993)^22^ (permission number # 5437641072307) by using CorelDraw X3 on enhanced Sentinel-2A MSI image (S2A_MSIL1C_20190220T081951_N0207_R121_T36RWT_20190220T102134, Copernicus Open Access Hub; https://scihub.copernicus.eu/dhus/#/home), processed using Envi 5.4 (Trial Version, https://www.l3harrisgeospatial.com/Software-Technology/ENVI).

The Abu Zenima area is one of Egypt's essential localities for sedimentary kaolinite resources. It has the highest quality and reserves of kaolinite in Egypt, which is estimated of about 120 million tons^18,19^. The lithological surface units at the Abu Zenima area are ranged from the Pre-Cambrian basement rocks to the Quaternary deposits (Fig. 1). The basement rocks are represented mainly by metamorphic schists, gneisses, and migmatites, in addition to older granitoids intruded by younger granites and sets of dykes with various compositions, which intruded sedimentary successions that accompanied different tectonic events^20–22^.

The sedimentary rocks are an interesting part of kaolinite exploration, especially those of the Carboniferous and Cretaceous ages^18^. The substantial outcrop of Kaolinite-bearing members in the study area belongs to the Matulla Formation of Upper Cretaceous (Santonian) age, which is exposed at the end of Wadi Khaboba and north of the El Markha plain (Fig. 1). This formation is composed of interbedded clays, marls, and sandstones of 60 m thick^23,24^. The current area was subjected to successive tectonics over the geological ages that left a series of faults of various trends (NNW–SSE, NE–SW, and E–W)^25^. Figure 1 shows the areal distribution of the lithological units in the study area.

### Remote sensing investigation

Multispectral (Sentinel-2A MSI) remote sensing data was used for mapping the Kaolinite-bearing formation; this product has 12-bit radiometric resolution that provides infrequent band saturation over highly reflective surfaces^26^. The Sentinel-2A MSI L1C datasets were acquired from the Copernicus Open Access Hub (https://scihub.copernicus.eu/dhus/#/home). It has 13 different spectral bands covering the visible and short wave infrared wavelength spectral region. The variation in spatial resolution for spectral bands has led to the use of this data in various applications (Table 1). The Sentinel-2A MSI L1C dataset is extracted from the MSI L1B product using radiometric and geometric correction methods. Table 1 shows the characteristics of the Sentinel-2A MSI spectral bands.

**Table 1:** Spectral bands of Sentinel-2 MSI sensor.

Sentinel-2 MSI		
Band	Wavelengths (nm)	Resolution (m)
1 (Coastal aerosol)	433–453	60
2 (Blue)	458–523	10
3 (Green)	543–578	10
4 (Red)	650–680	10
5 (RE 1)	698–713	20
6 (RE 2)	733–748	20
7 (RE 3)	773–793	20
8 (NIR)	785–900	10
8a (n NIR)	855–875	20
9 (Water vapor)	935–955	60
10 (Cirrus)	1360–1390	60
11 (SWIR 1)	1565–1655	20
12 (SWIR 2)	2100–2280	20

Sentinel-2A MSI data were preprocessed using the atmospheric correction method, made by Dark Object Subtraction (DOS1) algorithm that was presented from the Semi-Automatic Classification Plugin^27^ for QGIS version 7.6.1. All bands of the surface reflectance dataset were stacked and re-sampled to 10-m spatial resolution using the bilinear and a mean method; then, it was spatially subset to the area of interest by using SNAP (version 7.0).

**Figure 2:**
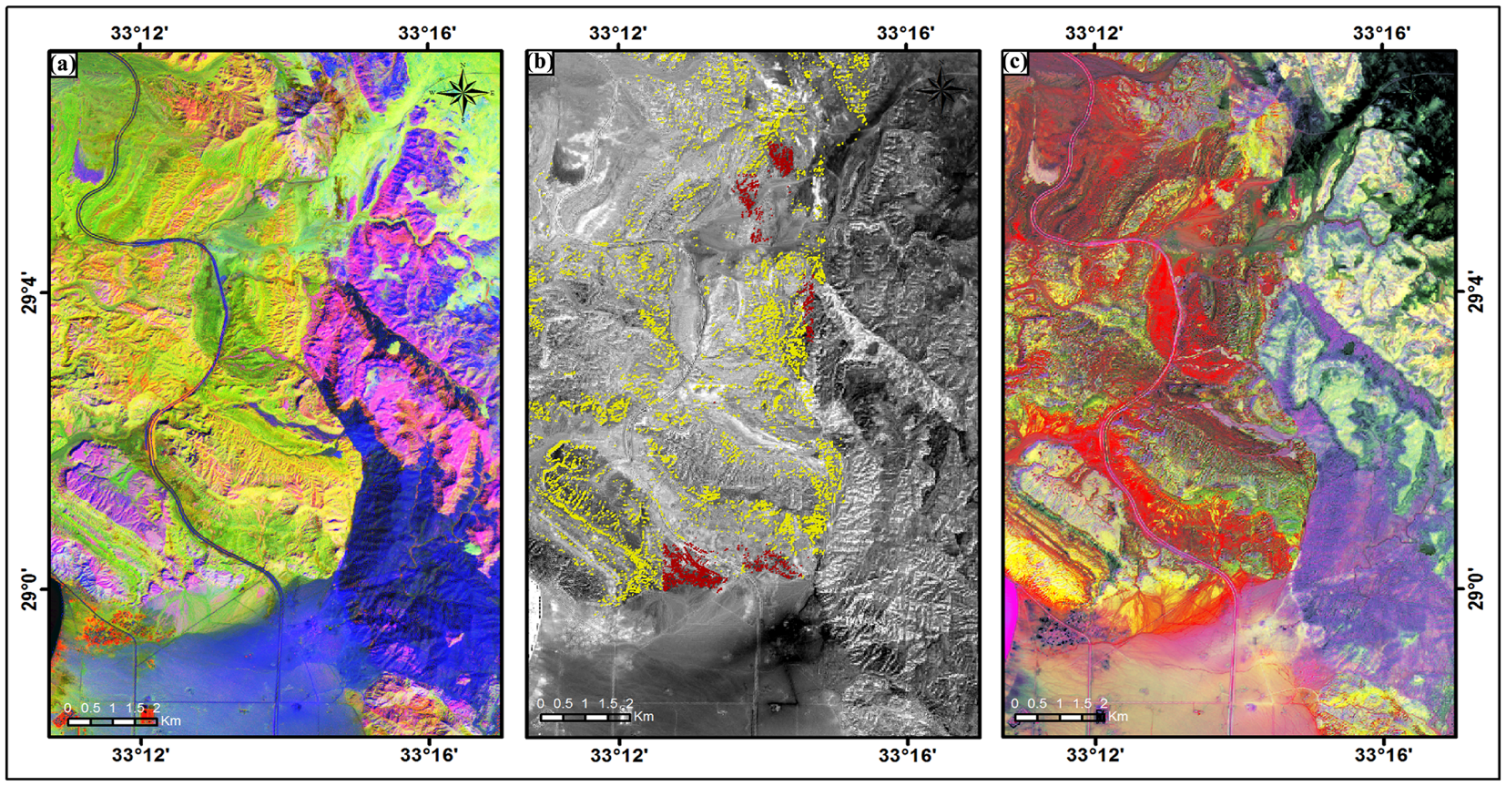
Illustrates (**a**) RGB color composite image of B8/B4, B4/B2, and B11/B12 ratios, (**b**) Kaolinite mineral index based on (B4/B8)*(3 + 4)/11 band ratio (red pixels refer to Matulla Formation and yellow pixels refer to the other clayey geological units), and (**c**) RGB color composite image of PC1, PC2 and PC3 indicates the occurrences of Kaolinite mineral (clay minerals: yellow pixels). These figures were generated from Sentinel-2A MSI image (S2A_MSIL1C_20190220T081951_N0207_R121_T36RWT_20190220T102134, Copernicus Open Access Hub; https://scihub.copernicus.eu/dhus/#/home) by using Envi 5.4 processing and analysis tools (Trial Version, https://www.l3harrisgeospatial.com/Software-Technology/ENVI).

Band-ratio (BR) and Principal Component Analysis (PCA) processing methods were used for particular kaolinite minerals mapping. Firstly, the band ratio technique was applied to display potential kaolin deposits by using ratios that emphasize spectral characteristics of significant surface material and suppress the others^28–31^. Kaolinite mineral shows a distinctive spectral reflectance response for visible and short wave regions of the electromagnetic spectrum^32^. The major absorption features of the kaolinite spectral curve occur around 1, 2, 10, and 12 bands based on different ion and ionic groups^33^. Consequently, the ratio combinations used in this study to discriminate Kaolinite-bearing formation are B8/B4, B4/B2, and B11/B12 as RGB (Fig. 2a). The boundary between sedimentary (yellowish-green and violet colors) and basement rocks (dark blue color) is delimited in the resultant BR image.

Because the Kaolinite-bearing formation is not clearly visible (very light purple color), the ((B4/B8) * (3 + 4)/11) ratio was used to focus on showing the kaolinite index better^34^. The kaolinite pixels were clipped for the Cretaceous age formation and presented over a greyscale image for straightforward clarification (Fig. 2b). Moreover, principal component analysis (PCA) was applied to Sentinel-2A MSI bands, which used the correlation between spectral bands and data variance calculation to confirm the delineation of the lithological units and Kaolinite-bearing formation. The three-high percentages of data variance PC1, PC2, and PC3 have proved influential in discriminating the occurrences of kaolinite mineral (yellow pixels) in the Matulla Formation (Cretaceous age) from the other clayey geological members (red pixels) in the study region (Fig. 2c). Obviously, the interesting formation has been observed in other scattered regions, such as in the north and the middle parts (Fig. 2).

Considering the previous geological map and the results of the processing steps on Sentinel-2A MSI, samples were taken from the Kaolinite-bearing (Matulla) Formation, specifically in the southern part of the studied region (symbol S; plotted in Fig. 1) in order to study their properties. This selected southern part is considered a guiding area for this formation in other occurrences.

### Field and laboratory investigation

#### Sampling and preparation of raw clay material

Ten representative clay samples were collected from the outcrop of Kaolinite-bearing members of Matulla Formation at Abu Zenima area, South Sinai, Egypt. The sampling was obtained from ten sites in the studied area (labeled as *AZ01-AZ010*). Each labeled sample (weighted about 15.0 kg) represents three sub-samples (weighted approximately 5.0 kg), collected from the uppermost, middle, and lower kaolinitic clay outcrop layer. Thereafter, the collected three sub-samples were mixed and quartered in order to ensure a representative and statistically valid clay sample at each site. Next, the collected samples were separately oven-dried at 60 ± 1 °C for 36 h. Next, each aliquot was manually crushed, and ground utilizing a laboratory agate ball mill and then passed through 63 μm sieves.

#### Characterization of raw clay material

The mineralogical analysis of the studied clay samples was assessed using XRD (Bruker D8 Discover diffractometer) with an accelerating voltage of 40 kV, current of 30 mA, and Cu Kα radiation wavelength (λ = 1.5418 Å). The scanning was conducted in a 2θ angle range from 3° to 80°. The XRF analysis was performed to get insight into the chemical composition of the studied clay samples (Philips PW 2400 WXRF Spectrometer). Fourier transform infrared (FT-IR) analysis of the studied AZ04 sample was executed using a Bruker Vertex-70 IR spectrometer (Germany) equipped with a rock-solid and diamond crystal interferometer at room temperature. The FT-IR spectra were obtained with 256 scans per sample at 4 cm^−1^ resolution in a wavelength range of 4000–400 cm^−1^. The particle surface morphology assessment of the studied AZ04 sample was examined using Scanning Electron Microscopy (SEM) at a magnification range of 5–20 kx with a 20 kV acceleration potential utilizing a JEOL JSM-6610LV SEM coupled with an energy dispersive X-ray spectrometer (EDX; Oxford Energy Dispersive X-Max 20 mm^2^). For the internal structural and morphological features of the studied AZ04 sample, a transmission electron microscope (TEM) was conducted at a magnification range of 20–50 kx (FEI, Tecnai G2 F20, 80–300 kV). The nitrogen adsorption/desorption measurements were also performed for the studied AZ04 sample in order to assess the specific surface area and pore size (Barrett-Joyner-Halenda (BJH), Langmuir, and Brunauer–Emmett–Teller (BET)) using a Micromeritics ASAP 2020 at 77 K. The thermal gravimetric (TG) and differential thermal (DTA) analysis were investigated using TGA PT 1000 (250/2500 μV, Linseis, Germany) with a heating rate of 10 °C /min ranging from the 30 °C up to 1000 °C. Moreover, the cation exchange capacity (CEC) of the studied AZ04 sample samples was estimated based on the ASTM C 837–81 method^5^.

The grain size analysis of clay samples was carried out using two methods based on the size fraction: wet-sieving and pipette-sedimentation methods^35,36^. The plasticity parameters were determined by Atterberg limits assessments (ASTM, D 4318-10), including plastic index (PI), plastic limit (PL), and liquid limit (LL). However, The LL and PL were performed based on the method of Casagrande^37^, and the difference between LL and PL was used to calculate the PI values (PI = LL – PL)^11,38^. The Index of Compositional Variability (ICV; Eq. 1) and chemical index of alteration (CIA; Eq. 2) were determined from the following equations:

1 $${\varvec{ICV}} = \left( {{\text{Fe}}_{{2{ }}} {\text{O}}_{3} + {\text{Na}}_{2} {\text{O}} + {\text{CaO}} + {\text{MgO}} + {\text{MnO}} + {\text{TiO}}_{2} } \right)/{\text{Al}}_{2} {\text{O}}_{3}$$

2 $${\varvec{CIA}} = 100 ({\text{Al}}_{2} {\text{O}}_{3} /({\text{Al}}_{2} {\text{O}}_{3} + {\text{ CaO}} + {\text{ Na}}_{2} {\text{O}} + {\text{ K}}_{2} {\text{O}})$$

The Bigot curves were obtained under room-temperature conditions by using an Adamel barelattograph. During drying, the linear drying shrinkage was derived from Bigot curves and drying sensitivity coefficient (DSC) was calculated according to the following equation: DSC = [(Water content of the plastic sample)—(Water content at constant shrinkage)]/(Water content at constant shrinkage)^35,39,40^. Briefly, the studied clay was crushed and rolled for a coarse grain size of 1 mm. The shaping of the clay requires a certain amount of water into pieces of dimension 15 × 15 × 30 mm (to measure the weight and length of wet pieces). These pieces were subjected to drying in open air conditions in the apparatus of Adamel Barellatograph. This device can track and trace the drying curve according to the mass loss. At the end of drying, the pieces were weighed and oven dried for 24 h at 110 °C to measure the final mass and dry lengths. These parameters allow measuring the drying shrinkage and water required for shaping, interposition, and colloidal.

## Results and discussion

### Characterization studies of the clay samples

#### XRD analysis

**Figure 3:**
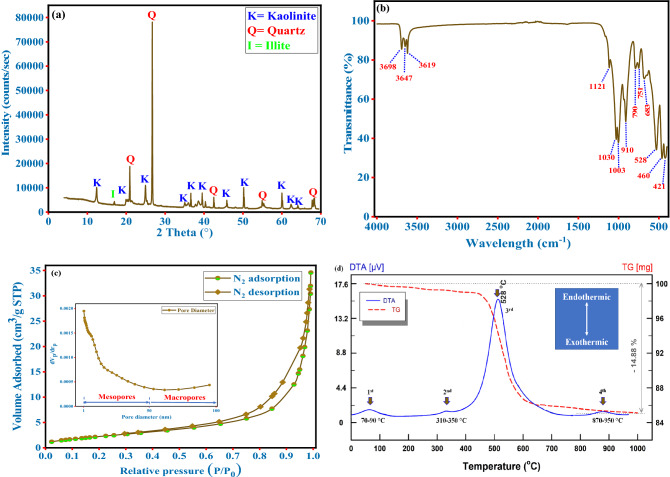
Shows the studied sample characterization: (**a**) XRD patterns, (**b**) FT-IR spectra, (**c**) N_2_ adsorption–desorption isotherm, and (**d**) DTA/TG curves.

The quantitative mineralogical analyses of the studied bulk clay samples from the outcrop of Kaolinite-bearing members of Matulla Formation at Abu Zenima area were performed and tabulated in Table 2. The obtained result indicated no significant differences between the collected clay samples in the mineralogical composition. Furthermore, the results show that kaolinite and quartz are the predominant constituents of all samples, with minor percentages of illite, hematite, and feldspar. The kaolinite amounts in the studied samples varied from 63.00 wt% in sample AZ08 to 71.00 wt% in sample AZ04. However, the quartz amounts in the studied samples ranged from 27.00 wt % as in sample AZ02 to 34.00 wt% in samples AZ05, AZ06, and AZ08 (Table 2). Minor amounts of illite (up to 2.00 wt%) were detected in most samples except samples AZ02, AZ07, AZ08, and AZ09. In addition, traces of hematite (up to 3.00 wt%) and feldspar (1.00 wt%) were also identified in a few samples. Figure 3a demonstrates the oriented XRD pattern of the selected representative sample (AZ04). In the representative sample, three main unique phases were detected: kaolinite, quartz, and illite. The prominent kaolinite peaks were identified and confirmed by the characteristic reflections at 2θ = 12.24° (001), 20.18° (multiple reflections), and 26.15° (002)^41^. According to Bragg’s law, the calculated interlayer spacing of main kaolinite reflections was found to be d_(001)_ = 0.72 nm and d_(002)_ = 0.34 nm. The main quartz reflections were found at 2θ = 20.9 and 26.6°, evidenced by the reported values in Table 2. Moreover, the small reflection at 2θ = 16.9° proved the existence of traces of illite. The presence of the aforementioned amounts of illite and kaolinite are favorable properties for ceramic use^42^. Figure S2 compares the XRD patterns of all studied samples (supplementary material).

**Table 2:** The mineralogical compositions of the studied bulk samples (wt%).

Sample No	AZ01	AZ02	AZ03	AZ04	AZ05	AZ06	AZ07	AZ08	AZ09	AZ10
Kaolinite	66	69	68	71	64	65	68	63	65	67
Quartz	28	27	31	28	34	34	29	34	33	31
Hematite	3	3	*Nd*	*Nd*	*Nd*	*Nd*	*Nd*	2	2	*Nd*
Illite	2	*Nd*	1	1	1	1	*Nd*	*Nd*	*Nd*	1
Feldspar	*Nd*	*Nd*	*Nd*	*Nd*	*Nd*	*Nd*	1	*Nd*	*Nd*	1

*Nd* not detected.

#### FT-IR analysis

Figure 3b shows FT-IR bands of the studied clay sample (AZ04). Several absorption bands are presented in this IR pattern, and these bands correspond to the vibration of various functional groups. Generally, kaolinite comprises four distinct hydroxyl groups^43^. The absorption bands of these four hydroxyl groups were detected at a wavelength range between 3500 and 3800 cm^−1^. These hydroxyl groups are absorbed water hydroxyl (3619 cm^−1^), inner-surface hydroxyl (3669 cm^−1^), inner hydroxyl (3647 cm^−1^), and outer-surface hydroxyl (3698 cm^−1^)^44^. Furthermore, the observed bands at 1121, 1030, and 1003 cm^−1^ were ascribed to the asymmetric stretching vibrations of Si–O bonds. However, the band at 528 cm^−1^ was attributed to Al–O–Si bond in kaolinite^41^. The bands at 910 and 790 cm^−1^ indicate the vibrations of Si–O–Si and the stretching vibration of Al–OH groups, respectively. Moreover, the band at 751 and 683 cm^−1^ was assigned to Si–O stretching bands^45^. The band at 528 cm^−1^ was attributed to O–Al–O bending and Al–O deformation^46^. The band at 460 cm^−1^ was assigned to Si–O bending/deformation bands. The Si–O–Si stretching band was observed again at 421 cm^−1^. The obtained FT-IR results and regarded bands confirmed the presence of kaolinite and quartz in the studied sample.

#### The clay surface measurements

Figure 3c illustrates the N_2_ adsorption–desorption isotherms of the studied clay sample (AZ04). According to the IUPAC classification, the studied clay sample follows an IV-type isotherm^47^. The IV-type isotherm is specifically characteristic of mesoporous materials, typical for materials with an average pore diameter of 2–50 nm. This result confirms the mesoporous character of the studied clay samples. Moreover, the H_3_ hysteresis loop was detected in the investigated sample, indicating the employment and evacuation of the mesopores by capillary condensation^43^. The results revealed that the investigated clay sample has BET surface area, total pore volume, and a mean pore diameter of 8.63 m^2^ g^−1^, 0.05 cm^3^ g^−1^, and 23.40 nm, respectively. Furthermore, the Langmuir surface area and the BJH surface area were found to be 6.64 and 11.01 m^2^ g^−1^, respectively. Additionally, according to the ASTM C 837-81 method, the CEC of the investigated sample was found to be 11 meq/100 g. Furthermore, in order to get insight into the surface charge of the studied clay sample, the point of zero charges (pH_pzc_) was determined as shown in Figure S1 (Supplementary Material). The pH_pzc_ was determined by the pH drift method, as reported perviously^48,49^, and found to be 5.31 for sample AZ04. Determining pH_pzc_ is crucial for the ceramic industry in case chemical modifications are used for the raw clay material.

#### Thermal characteristics

The thermal gravimetric and differential thermal analysis (TG–DTA) analysis were conducted in order to get insight into the physicochemical variations throughout the endo and exothermic effects. Figure 3d illustrates the TG–DTA curves of the investigated clay sample at a temperature range of 30–1000 °C. Figure 3d demonstrates a considerable weight loss in the AZ04 sample with increasing the heating temperature. From the DTA curve, four successive endothermic peaks have appeared. However, no exothermic peak could be detected along with the temperature range. The first endothermic peak was observed at a temperature range from 70 to 90 °C. This endothermic peak could be ascribed to the elimination of hydroscopic water or dehydration of interlayer water of the existing clay minerals (Fig. 3d). The weight loss for the first endothermic peak at this temperature was found to be 2.1%. With increasing the temperature, the second endothermic peak was detected at a temperature range from 310 to 350 °C. The endothermic peak at this temperature range is characteristic of goethite. At the second endothermic peak, the mass loss was about 1.0%. The third endothermic peak, a broad endothermic peak was appeared and centered at a temperature value of 528 °C (Fig. 3d). The appearance of this large peak could be attributed to the dehydroxylation of structural OH from the kaolinite. More specifically, this dehydroxylation happened through restructuring the octahedral layer of kaolinite in atetrahedral configuration in-metakaolinite^50^. Additionally, the conversion of $\alpha$–quartz to $\beta$–quartz phase is also occurred in the same temperature range^51^. The corresponding weight loss for this endothermic peak was found to be 11.7%. The last endothermic peak was detected at 870 to 950 °C (Fig. 3d). The existence of this peak confirms the presence of well-crystallized kaolinite, and this endothermic peak is due to the breakdown of metakaolinite^52^. The obtained result was realized to agree with the XRD result.

#### The morphological analysis

**Figure 4:**
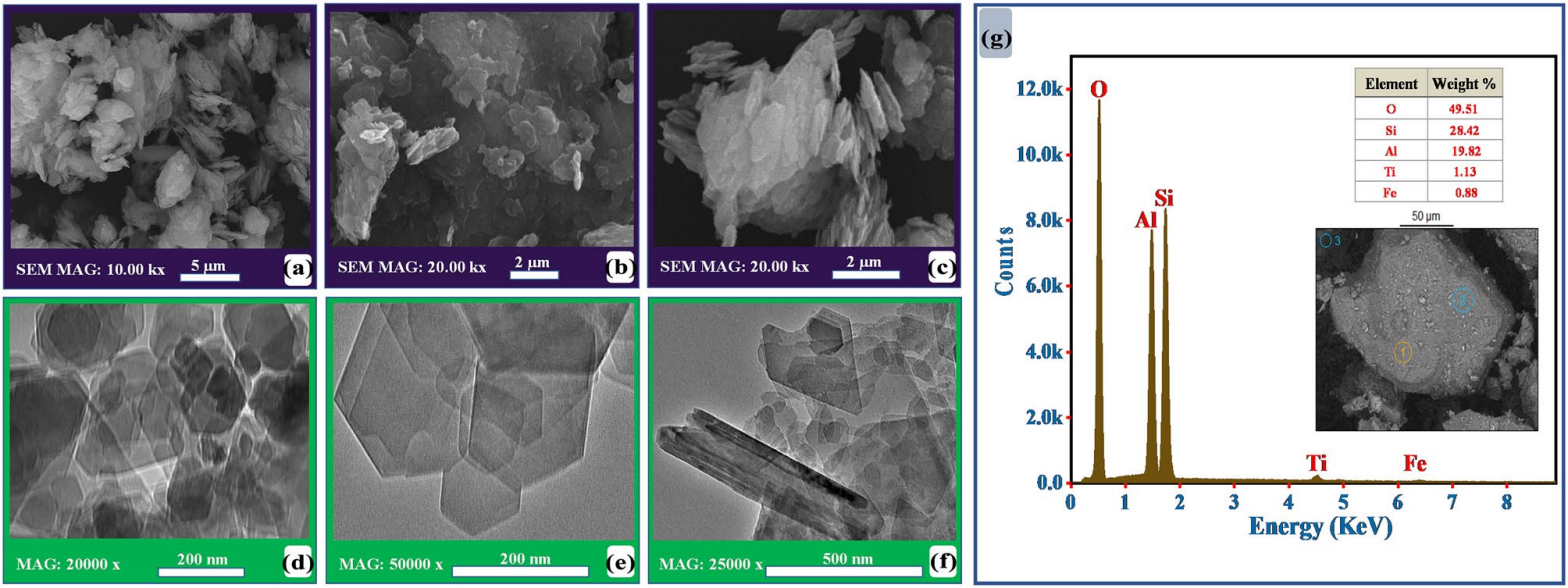
Shows the morphological analysis of the studied clay sample: (**a–c**) SEM micrographs, (**d–f**) TEM images at different magnifications, and (**g**) EDX spectra.

 SEM, TEM, and EDX were performed on the studied clay sample to get insight into the surface morphology, internal structure, and composition, respectively. Figure 4a–c illustrates the SEM micrographs for the studied sample (AZ04) at different magnifications. The kaolinite surface appeared with a complex morphology showing randomly arranged small platelets ranging from 0.5 to 2.0 μm (Fig. 4a). On the other hand, some kaolinite particles showed hexagonal corners and edges in their structure (Fig. 4b). Moreover, kaolinite in the studied sample presented randomly scattered dislocations of particles in stacked layers (Fig. 4c). These dislocations could occur along the Y-axis and multiples of b0/3^46^. Figure 4d–f demonstrates the TEM images of AZ04 at different magnifications. The TEM analysis was conducted to disclose the internal structural features of the investigated sample. The kaolinite presented well-crystallized and well-constituted particles displaying a typical hexagonal and euhedral morphology (Fig. 4d,e). Furthermore, large units were observed as booklets or platey morphology (Fig. 4f). These results indicate the pseudo-hexagonal structure of kaolinite layers. The kaolinite particles are mostly anisometric with a tiny thickness (along the Z-axis) compared to their other dimensions. The chemical constituents on the surfaces of the clay sample were assessed by EDX analysis (Fig. 4g). The obtained results revealed that the most abundant constituents in the studied sample were O, Si, and Al. In addition, minimal amounts of Ti and Fe were also detected. The EDX results confirmed the XRD and chemical composition analysis, demonstrating that kaolinite and quartz are the predominant constituents in the studied clay sample.

#### Geochemistry analysis

**Figure 5:**
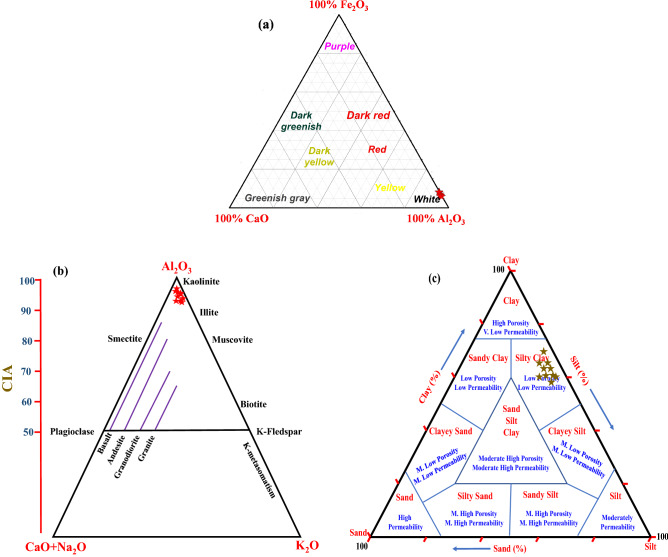
Illustrates: (**a**) The three-component diagram showing fired brick color differences (after (Piltz (1964)^56^), (**b**) CIA ternary diagram (after Nesbitt and Young (1982, 1984)^63,64^), (**c**) Textural classification of studied clay sediments following the relation between sand, silt and clay components and their controls over porosity and permeability (after Shepard (1954)^70^).

The chemical composition analysis of the investigated bulk samples was conducted and is presented in Table 3. The obtained results revealed that SiO_2_, Al_2_O_3_ were the most abundant oxides in all samples, along with traces of the other oxides as listed in Table 3. The findings of SiO_2_, Al_2_O_3_ amounts were in agreement with the mineralogical composition of the investigated clay samples. The existing major oxides (SiO_2_ and Al_2_O_3_) were predominantly associated with existing clay minerals and quartz in the samples (confirmed by XRD) (Table 2). However, in small quantities, the other oxides such as MgO, K_2_O, CaO, Na_2_O, and P_2_O_5_ were present in all samples. The values range of SiO_2_, Al_2_O_3,_ and Fe_2_O_3_ in the studied samples were found to be 53.28–56.07, 26.84–28.43, and 1.45–3.51 wt%, respectively. The values of these three oxides confirm that these clays are desirable for ceramic products^53^. Moreover, the quartz content in the studied samples is tolerable since these amounts can be simply digested using the vitreous flow during firing^42^. Generally, Fe_2_O_3_ (total iron oxide) is the primary colorant component in clayey materials. Moreover, the existing amounts of Fe_2_O_3_ are sensitive to firing (responsible for the reddish color after firing) and usually generate unexpected results in the texture and color of fired clays^54,55^. The obtained results showed high Fe_2_O_3_ content in samples AZ01, AZ02, AZ08, and AZ09 (Table 3). These results could be due to the presence of hematite and illite minerals in the aforementioned samples (Table 2). Nevertheless, the Fe_2_O_3_ content in clay is a responsible factor for the ceramic wares coloration and the presence of other constituents such as TiO_2_, MgO, CaO, MnO and can considerably influence the color of fired clays^52^. Additionally, other factors can influence the color of fired clay products, such as firing temperature, furnace atmosphere, and Al_2_O_3_ content in the clay. According to Piltz (1964), the oxides (Al_2_O_3_, CaO, and Fe_2_O_3_) importance for color to 100% were calculated, and the ternary diagram was plotted showing the color of the investigated clay samples (Fig. 5a)^56^. Moreover, the negative correlation between Al_2_O_3_ and either TiO_2_ or Fe_2_O_3_ in the studied samples (Table 3) suggests that Fe and Ti could be substituted for Al in the kaolinite structure in the clayey samples. The existence of CaO and MgO (earth-alkaline oxides) content was low, with values of less than 0.13 and 0.38 wt%, respectively (Table 3). These content values indicated the absence of carbonates (non-calcareous clays) in the samples^57^. The low content of earth-alkaline oxides is significantly helpful in preventing the shrinkage of raw brick^58^. Furthermore, the amount of alkaline oxides such as K_2_O and Na_2_O in the investigated clay samples was relatively low (less than 0.24 wt%) (Table 3). In fact, the alkaline oxides (i.e., K_2_O and Na_2_O) are acting as flux materials, the kaolinitic clays naturally have a relatively low amount of flux oxides^52,59^. The flux oxides (2.49–3.36 wt%; Table 3) such as TiO_2_, Fe_2_O_3_, K_2_O, MgO, and CaO are important during firing, which assists the melting of silicates and also binds the clay particles together^58^. The loss on ignition (LOI) values for the studied samples were found to be in a range of 11.36–14.50 wt% (Table 3). These obtained LOI values could be attributed to the presence of organic matter, substantial volatiles, dehydroxylation of the clay minerals, and/or decomposition of carbonates. These findings were found to be in agreement with other researchers when they studied various types of clays^57,60–62^. These results were previously confirmed by thermal analysis of the studied samples as shown in Fig. 3d.

**Table 3:** The chemical composition of major oxides (wt%), loss of ignition, and alteration indices of the studied bulk samples.

Sample no.	AZ01	AZ02	AZ03	AZ04	AZ05	AZ06	AZ07	AZ08	AZ09	AZ10
SiO_2_	55.42	53.87	54.05	56.07	53.28	54.73	55.11	54.58	54.26	54.64
Al_2_O_3_	26.84	27.45	28.43	29.01	27.46	28.35	27.05	27.26	28.18	28.76
TiO_2_	1.32	0.52	1.68	1.71	1.82	1.84	1.77	0.96	0.71	1.73
Fe_2_O_3_^TT^	2.02	2.89	1.86	1.45	2.36	1.77	2.37	2.83	3.51	2.55
MgO	0.28	0.34	0.32	0.36	0.37	0.31	0.34	0.32	0.33	0.38
CaO	0.08	0.11	0.09	0.11	0.05	0.07	0.12	0.13	0.06	0.08
K_2_O	0.15	0.12	0.11	0.21	0.19	0.17	0.15	0.18	0.17	0.19
Na_2_O	0.24	0.22	0.19	0.23	0.18	0.17	0.23	0.17	0.22	0.21
P_2_O_5_	0.04	0.05	0.03	0.03	0.04	0.04	0.03	0.06	0.05	0.04
LOI	13.25	14.50	12.71	10.35	13.73	12.42	12.47	13.64	12.22	11.36
Total	99.64	99.62	99.47	99.53	99.48	99.87	99.64	99.86	99.71	99.94
SiO_2_/Al_2_O_3_	2.06	1.96	1.90	1.93	1.94	1.93	2.04	2.00	1.93	1.90
Flux	2.77	2.68	2.87	3.36	3.15	2.49	3.21	2.63	3.29	3.41
CIA	98.28	98.39	98.65	98.07	98.49	98.57	98.19	98.27	98.43	98.36
ICV	0.15	0.15	0.16	0.18	0.18	0.15	0.18	0.16	0.18	0.18
CEC	10.00	11.00	11.00	11.00	10.00	11.00	10.00	9.00	10.00	10.00

*TT* total iron as Fe_2_O_3_, *LOI* loss on ignition, *CIA* chemical index of alteration, *ICV* index of composition variability, *CEC* cation exchange capacity.

Originally, the mass ratio of SiO_2_/Al_2_O_3_ found in pure kaolinite and montmorillonite was 1.18 and 2.36, respectively^11^. The SiO_2_/Al_2_O_3_ ratio in the studied clayey samples was found in the range of 1.90–2.06. The obtained values are higher than the original value of pure kaolinite and lower than montmorillonite, confirming the presence of quartz in the studied clayey samples.

Furthermore, two different indices, namely the chemical index of alteration (CIA) and the index of composition variability (ICV), were utilized to deduce the source rock and paleo-weathering of the studied clay samples. The CIA, initially proposed by Nesbitt and Young (1982, 1984), was commonly used to classify the degree of chemical weathering of source rocks^63,64^. The CIA values of the investigated clay samples were found to be in a range from 98.07 to 98.65. The values imply that all clay samples from the studied area were subjected to intensive chemical weathering conditions (Table S1). Also, the intensive weathering conditions led to an enrichment of Al-rich products. The high CIA values indicated the maturity of Abu Zenima clay deposits and confirmed that these clay deposits contained residual clays rich in kaolinite (Fig. 5b)^65^. The ICV, initially proposed by Cox et al. (1995), was utilized to measure the compositional maturity and abundance of alumina compared to the other existing major cations in the studied samples^66^. When ICV $\ge \hspace{0.17em}$1.0, these values indicate compositionally immature mud rocks and the presence of a high amount of non-clay silicate minerals. On the contrary, when ICV $<$ 1, this reveals that the sample is mostly clay minerals and compositionally mature. For the studied samples, the ICV values ranged from 0.15 to 0.18. The very low ICV values confirm that the samples were mostly kaolinite accumulated in cratonic environments and the area was tectonically quiescent^67,68^.

### Ceramic properties and technical characteristics

#### Granulometric study

The particle-size distribution of clayey materials is a substantial factor in evaluating their suitability for several applications, especially in the ceramic industry. In fact, the particle-size distribution of clayey materials plays a crucial role during the drying and firing process in characterizing the properties of suspensions and green pastes (i.e., viscosity and plasticity)^69^. In this regard, the granulometry analysis was performed to get insight into various particle size and their quantity (wt%) for the studied clay samples (Table 4). The obtained results of granulometry analysis showed that the three main size categories (sand, silt, and clay) were present in all studied clay samples. The clay fraction (particles < 2 µm) was the most predominant size in the studied samples and was found in the percentage range of 47.7–61.7%. The existence of clay fractions with high values is favorable for the ceramic industry. The silt fraction (particles range of 2–60 µm) was found to be the second-highest percentage (26.8–38.8%) after the clay fraction (Table 4). A small amount of the sand fraction (particles ˃ 60 µm) was also detected and found in a range of 10.4–13.7%. Overall, the analyzed clay samples exhibit a low variation range in particle size distribution. However, the coarse sand fraction (particles ˃ 60 µm) is the most considerable problem for the ceramic industry. This problem can be simply solved by grinding and sifting, and after that becomes suitable for ceramic products. The obtained results from the particle-size analysis were further plotted in the ternary diagram of Shepard (1954) (Fig. 5c)^70^. Shepard's classification suggests that the studied clay sample could be silty clay. Additionally, the ternary diagram also assessed the relationship between clay, silt, and sand fraction and their controls over permeability and porosity (Fig. 5c). According to the classification of Shepard, the investigated samples were plotted in the low porosity and low permeability fields (the same classification domain). The porosity and permeability of the studied samples were also confirmed by the classification and interpretation of McManus (1988)^71^.

**Table 4:** The consistency limits (%) and Particle-size distribution of the studied clay samples.

Sample no.	AZ01	AZ02	AZ03	AZ04	AZ05	AZ06	AZ07	AZ08	AZ09	AZ10
**Consistency limits (%)**										
Liquid limit (LL)	54.0	52.0	47.0	40.0	42.0	41.0	44.0	43.0	48.0	45.0
Plastic limit (PL)	25.0	24.0	27.0	26.0	27.0	27.0	28.0	24.0	28.0	26.0
Plasticity index (PI)	29.0	28.0	20.0	14.0	15.0	14.0	16.0	17.0	20.0	19.0
**Particle-size distribution (%)**										
Sand (˃60 µm)	11.5	10.9	12.3	10.4	13.5	12.9	12.2	13.1	11.8	13.7
Silt (2–60 µm)	26.8	34.2	30.5	32.7	38.8	31.0	33.2	34.9	33.1	31.5
Clay (< 2 µm)	61.7	54.9	57.2	56.9	47.7	56.1	54.6	52.0	55.1	54.8

#### Evaluation of plasticity

**Figure 6:**
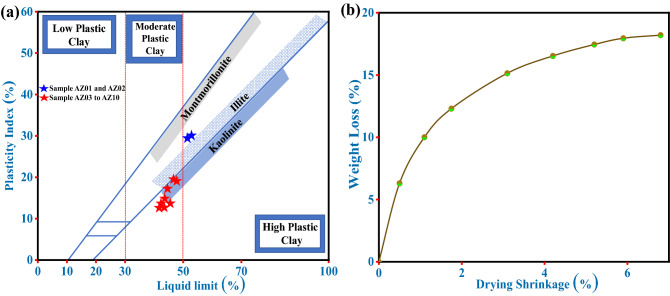
Shows: (**a**) The position of studied clay samples on the Holtz and Kovacs diagram (after Holtz and Kovacs (1981)^74^) and (**b**) Bigot curves of the clay sample.

Plasticity is considered the most crucial parameter for producing traditional ceramics and manufacturing clayey products. Plasticity provides the needed information about the workability of any material under stress without breaking and the influence on the produced shape after releasing this stress. In other words, plasticity can offer information about utilizing mechanical properties and pressure in producing clay bodies and ceramics^72^. The plasticity of clayey materials can be influenced by their particle-size distribution, morphology, the origin of geological formation, mineralogical composition, and impurities (i.e., organic matter and non-clay minerals)^54^. Additionally, the plasticity of clay is directly proportional to its water content, especially for platy-like clay minerals^73^. Table 4 demonstrates the consistency limits of all investigated samples, including LL, PL, and PI. The LL and PL values of the investigated clay samples were found in the range of 40.0–53.0% and 24.0–28.0%, respectively. Consequently, the PI values were found in the range of 14.0–28.0%. The obtained results revealed that the existing clay fraction in the sample could be a significant factor that influences the plasticity, in addition to mineralogical composition such as quartz content. These findings were found to be in agreement with other authors when they studied the plasticity of other clay deposits^42,72^. Thereafter, the calculated consistency limits (LL and PI) values (%) were plotted on the diagram of Holtz and Kovacs (1981) (Fig. 6a)^74^. This diagram was initially constructed to determine the clay materials' position in the three plasticity levels. According to Holtz and Kovacs diagram, the studied clay deposits fall into the moderate plastic clay region except for samples AZ01 and AZ02 (blue star), which are found in the high plastic clay region (Fig. 6a). The location of these samples (AZ01 and AZ02) could be ascribed to the high LL percentage and amount of clay (particles < 2 µm) and silt (particles range of 2–60 µm) fractions that existed in these samples. Generally, the obtained results in Table 4 showed that PI values are significantly affected by LL values variations. The LL values of the investigated samples were found within the acceptable LL range (30–60%; based on the ceramic literature) for ceramic production^69^. On the other hand, for ceramic applications, high PL values of clayey material can be responsible for production difficulties such as drying, grinding, and firing stages. Also, greater mechanical strength is always associated with higher plasticity materials^53^. Overall, the evaluated consistency limits of the investigated clay samples confirm that most of these clay deposits are suitable for various ceramic applications.

#### Bigot curves

In order to get insight into the behavior of the investigated clay deposits when dried and also to evaluate the clay's capacity to expel or retain water, Bigot’s curve was performed. Bigot's curves are useful characterization tools commonly used in clay-based ceramic manufacture to examine the ability of clays and pastes to dry^69^. Moreover, Bigot's curves are a helpful tool or preliminary indicator for selecting suitable materials for ceramic products. Figure 6b illustrates Bigot’s curve of the investigated clay sample (drying shrinkage vs. weight loss). The obtained result from Bigot’s curve, drying at room temperature, implies that the studied sample contains about 6.47% moisture water. Furthermore, the clay sample's maximum drying shrinkage was 6.81%, indicating a moderate drying character.

### Suitability of raw clay material for industrial applications

**Figure 7:**
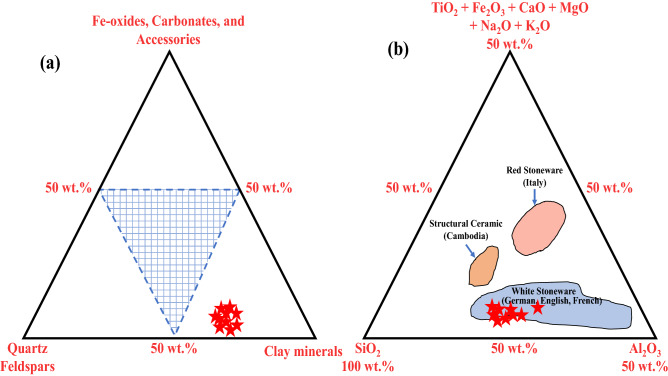
Illustrates: (**a**) Ternary diagram of Fiori et al. (1989)^75^ for classification of the ceramic-based clay bodies based on the mineralogical parameters and (**b**) Triangular diagram of clay-rich materials of Fabbri and Fiori (1985)^76^ based on the geochemistry data.

Based on the mineralogical composition, particle-size distribution, chemical analysis, and material characterization, the suitability assessment of the investigated raw clay material can be performed via various plotting diagrams. Fiori et al. (1989) constructed a ternary diagram based on the mineralogical parameters (clay minerals, quartz + feldspars, and the total of other existing components (Fe-oxides, carbonates, and accessories)) of the targeted clayey material (Fig. 7a)^75^. These diagrams are considered a practical classification of ceramic-based clay bodies and are a beneficial reference for tile production. The plotted data of the studied samples were found in rich clay minerals. According to the criteria of Fiori, the presence of a high amount of clay fraction in the sample makes it suitable for white ceramic stoneware and clinker (due to the high amount of kaolinite component)^75^. The mineralogical and chemical composition of the studied clay deposits were found to be close to the standard Malaysian clay (BBC), which is commonly used as a raw material in ceramic tiles production^60^. This property closeness confirms that the investigated clay of the Abu Zenima area can also be used in ceramic tiles manufacturing. Furthermore, another ternary diagram was created by Fabbri and Fiori (1985) utilizing the geochemistry data (silica, alumina, and some other oxides) of the investigated samples in order to classify clay materials and industrial ceramic bodies (with some countries' ceramic references)^76^. Figure 7b depicts the plotting of the obtained data on the ternary diagram compared with the reference clay of some countries' industrial ceramic bodies. The diagram revealed that the studied samples were plotted in the white stoneware field which referenced German, English, and French industrial ceramic bodies. These samples were unsuitable for red stoneware (Italy) or structural ceramic (Cambodia) products. The plotting results in Fig. 7b confirm the previously obtained results from the ternary diagram of Fiori et al. (1989)^75^. The inappropriateness of the studied clay deposits in red ceramics or red stoneware manufacturing can be explained based on the assumptions of Fiori et al. (1989) and Murray (2006)^75,77^. Based on these assumptions, clayey material containing Fe_2_O_3_ amount ≥ 5.0 wt% is classified as red-firing clays (Table 3). However, the clays that comprise Fe_2_O_3_ amount of between 5.0 and 1.0 wt% are B tan-burning clays, and those with Fe_2_O_3_ amount < 1.0% are white firing clays. Due to the average amount of Fe_2_O_3_ (1.77–3.51 wt%), the studied clays are not recommended for the production of red ceramics.

**Figure 8:**
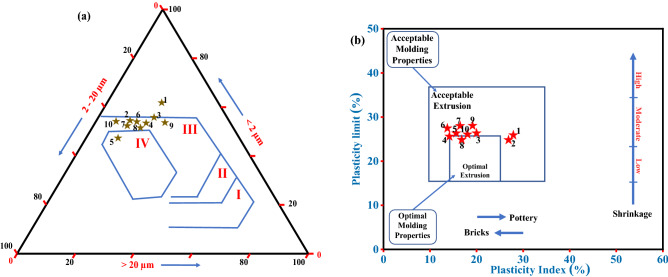
Shows: (**a**) Grain size classification of the clay-rich materials according to Winkler's scheme (after Winkler (1954) and Nyakairu et al. (2002)^78,79^); Fields are defined as: (I) common bricks, (II) vertically perforated bricks, (III) roofing tiles and masonry bricks, and (IV) hollow products, and (**b**) Molding prognostic through the Atterberg limits (workability chart) (after Bain and Highley (1979) and Hosni et al. (2021)^72,80^).

The appropriateness of raw clay material for different ceramic goods can be determined based on the results of the particle-size analysis. Figure 8a depicts the plotting of the obtained data on Winkler's diagram^78^. The Winkler's chart, developed by Nyakairu et al. (2002)^79^, evaluated the suitability of the clay-rich material based on ceramic products in three groups of grain fractions (< 2.0 µm, 2.0–20.0 µm, and > 20.0 µm). The diagram revealed that all the studied samples are suitable for roofing tiles and masonry bricks except samples AZ01 and AZ05 (Fig. 8a). The AZ05 was found to be more suitable for hollow products, which could be attributed to the higher 2.0–20.0 µm fractions compared to the other samples. However, sample AZ01 was found not favorable for product specifications in Winkler's diagram (Fig. 8a), which would require a beneficiation process before its utilization.

Furthermore, Bain and Highley (1979) constructed the clay workability chart based on the plasticity index and plastic limit of the investigated clays (Fig. 8b)^80^. According to the clay workability chart, most of the investigated clay materials were located in the acceptable extrusion field except for sample AZ08, which was found in the optimal molding region. This finding suggests that the investigated clay samples could be used in pottery and bricks. According to Bain and Highley (1979)^80^, the clay samples located outside both the acceptable and optimal extrusion region, not in the present work, could be suitable and utilized for hand wheel-throwing and/or soft-mud working.

An important factor influencing raw materials' suitability in the industrial sector is the closeness and accessibility of the expected raw material accumulations. The investigated clay deposits outcrop at Abu Zenima area, specifically of Matulla Formation, are easily accessible from the new main Suez-Sharm El-Sheikh asphaltic road. The site is located at 130, 210, and 280 km from Suez, Ain El-Sokhna, and Cairo cities, respectively. The closeness of the investigated area to most of the industrial areas significantly increases the suitability of our explored clay deposits.

## Regional significance of the present study

Due to the instant development and national construction projects, the Egyptian ceramic industry has significantly evolved, and the demand for ceramic industry raw materials has rapidly increased. Nowadays, Egypt is one of the prominent African ceramic tile producers and 10th globally with a production value of about 300 million m^2^/yearly (mordorintelligence.com). Additionally, ceramic manufacturing, including industrial, domestic, and building products, is one of the most prosperous industries in the Egyptian economy. Moreover, the increased expansion and spending on infrastructure, housing, and urbanization are expected to increase per capita ceramic consumption in the upcoming years to higher levels. Therefore, the exploration of new reserves of clay deposits as raw materials is thus required to meet the increased demand of clay-consuming industries. Since there are significant reserves of clay deposits in the Abu Zenima area, South Sinai, Egypt, these deposits have not been clearly studied and characterized according to their mineralogical, chemical, and technological properties for the ceramic industry. Furthermore, ceramic manufacturers in the investigated areas are expected to bring steady producers in the ceramic industry in the long term in order to gain the advantage of low-cost raw materials, labor, and factory construction. The kaolin deposits are the main and most expensive component in ceramic manufacturing. Thus, the existence of high kaolin reserves in the studied area with reasonable quality and quantity would significantly help reduce the manufacturing cost and overwhelm the high consumption rate. The major chemical composition and kaolinite content of the investigated kaolin was compared with several reported Egyptian kaolin deposits in the literature and tabulated in Table 5. The raw materials presented in this study demonstrated the most suitable raw materials for various types of ceramic industry based on their natural composition. The suitability of the studied kaolinitic clay for industrial applications was proven by geochemistry, granulometric, ceramic, and technical characteristics. Therefore, the findings of the present investigation will help to improve knowledge of the kaolin clay of the Abu Zenima area as well as contribute to the exploitation of these deposits and optimization of ceramic fabrication.

**Table 5:** Comparison of the main chemical (wt%) and mineralogical (wt%) composition of the investigated kaolin and varied kaolins in different localities from Egypt from literature.

Locality	Al_2_O_3_	SiO_2_	Fe_2_O_3_	TiO_2_	Kaolinite
El-Tieh plateau (Sinai) (El-Naggar 2014)	32.40	51.13	2.06	1.40	≈ 90.00
El-Tieh plateau (Sinai) (Morsy et al. 2014)	36.78	47.87	0.41	2.20	≈ 98.50
Kalabsha (S. Aswan) (Saber et al. 2018)	36.08	40.95	5.34	3.24	≈ 99.00
Kalabsha (Aswan) (Baioumy and Gilg 2011)	33.94	49.76	0.81	3.01	84.65
Abu Darag area (Red Sea) (Baioumy 2014)	28.70	55.74	1.49	2.11	83.57
F.El-Gozlan (Sinai) (Abdel-Khalek 1999)	35.21	44.43	0.92	1.38	92.43
Gabal El Gunna (Sinai) (Hassaan et al. 2015)	33.41	49.24	0.33	1.45	85.32
Wadi El Hamadiya (Sinai) (Drweesh et al. 2016)	34.74	47.97	1.46	2.26	89.00
Wadi Hagul (Suez) (Bahgaat et al. 2020)	19.96	48.58	16.10	2.77	–
Current Study (Average)	27.88	54.60	2.36	1.41	66.60

## Conclusions

This study included a detailed study of clay deposits (kaolinite-rich clay) belonging to the Matulla formation in the Abu Zenima area utilizing remote sensing data, mineralogical, and geochemical analysis. The remote sensing processing techniques on Sentinel-2A MSI data (BR and PCA) allow better accuracy in Kaolinite-bearing Formation classification and lithological identification on a regional scale. The obtained data from remote sensing indicated that the investigated area is considered promising due to the presence of clay deposits in large quantities. The Upper Cretaceous (Santonian) clay deposits (ten samples) at the Abu Zenima area were extensively evaluated using several characterization techniques. The mineralogical analysis (XRD) revealed that kaolinite and quartz were the predominant constituents of the studied clay samples, associated with minor percentages of illite, hematite, and feldspars. The surface measurements revealed that the representative sample has a BET surface area, total pore volume, and a mean pore diameter of 8.63 m^2^ g^−1^, 0.05 cm^3^ g^−1^, and 23.40 nm, respectively, and follows an IV-type isotherm (mesoporous characteristic). The chemical analysis of the raw clay powders confirmed that Al_2_O_3_ and SiO_2_ were the most abundant oxides in all samples. The high CIA values indicate the maturity of Abu Zenima clay deposits and confirm that the studied clays are rich in kaolinite. From a technological point of view, the studied samples are largely suitable for various ceramic products. The clay fraction with high values is favorable for the ceramic industry. Moreover, the clayey materials demonstrated moderate plasticity that is suitable for white ceramic stoneware and clinker. Comparing the mineralogical and chemical composition of the studied clay deposits to the standard Malaysian clay showed high similarity and confirmed its suitability to be used as a raw material in ceramic tiles production. The results also revealed the inappropriateness of these clays in red ceramics or red stoneware manufacturing due to the average amount of Fe_2_O_3_. According to Winkler's specifications, the studied samples are suitable for roofing tiles and masonry bricks, except for samples AZ01 and AZ05, which are suitable for hollow products. Sample AZ01 could be treated with a beneficiation process to render it more convenient for the products mentioned above. Furthermore, the workability of the studied clay materials was found to be in the acceptable extrusion field, and sample AZ08 was found in the optimal molding region. Overall, the obtained positive results and promising technological findings verify the potential of Upper Cretaceous (Santonian) clay deposits as possible raw materials for white ceramic stoneware, tiles, and brick products in addition to their closeness and easy accessibility for the expected industrial sector.

## Supplementary Information

Supplementary Information.

## Data Availability

The datasets used and/or analysed during the current study available from the corresponding author on reasonable request.
