# Supplementary material for: Evaluation insight into Abu Zenima clay deposits as a prospective raw material source for ceramics industry: Remote Sensing and Characterization
Source: Sci Rep. 2023 Jan 2;13:58. doi: 10.1038/s41598-022-26484-5 (PMC9807103; doi:10.1038/s41598-022-26484-5)
Supplement: Supplementary file 1 — Supplementary Information. [file 41598_2022_26484_MOESM1_ESM.pdf]

Evaluation Insight into Abu Zenima Clay Deposits as a Potential Raw Material Source for Ceramics Industry

Ali Maged[1,*], Sherif Ahmed Abu El-Magd[1], Ahmed E. Radwan[2,3], Sherif Kharbish[1], Sara Zamzam[4]

[1] *Geology Department, Faculty of Science, Suez University, El Salam City, P.O. Box 43518, Suez Governorate, Egypt*

[2] *Faculty of Geography and Geology, Institute of Geological Sciences, Jagiellonian University, Gronostajowa 3a, 30-387, Kraków, Poland*

[3] *Exploration Department, Gulf of Suez Petroleum Company, Cairo, Egypt*

[4]*Department of Geology, Faculty of Science, Zagazig University, Zagazig City, Sharkia Governorate, 44519, Egypt*

*Corresponding authors: Ali Maged (Ali.Maged@suezuni.edu.eg)

Table S1. The degree of chemical weathering based on the chemical index of alteration (CIA).

CIA ranges	Weathering degree
**50-60**	Weak degree of chemical weathering
**60-80**	Moderate degree of chemical weathering
**80-100**	Intensive degree of chemical weathering

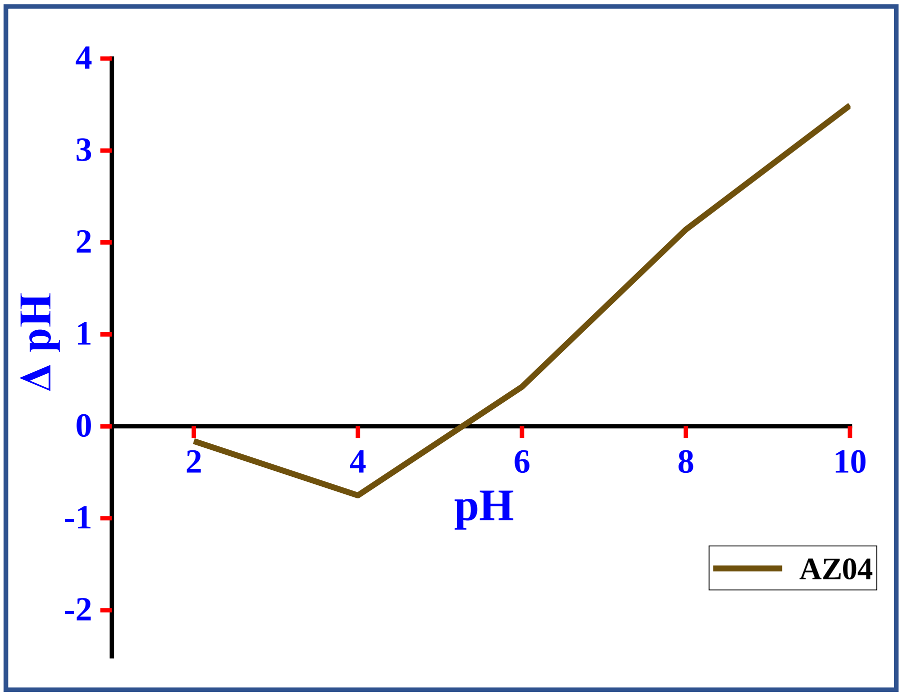

Figure S1. The pHzpc measurements of the studied clay sample (AZ04).

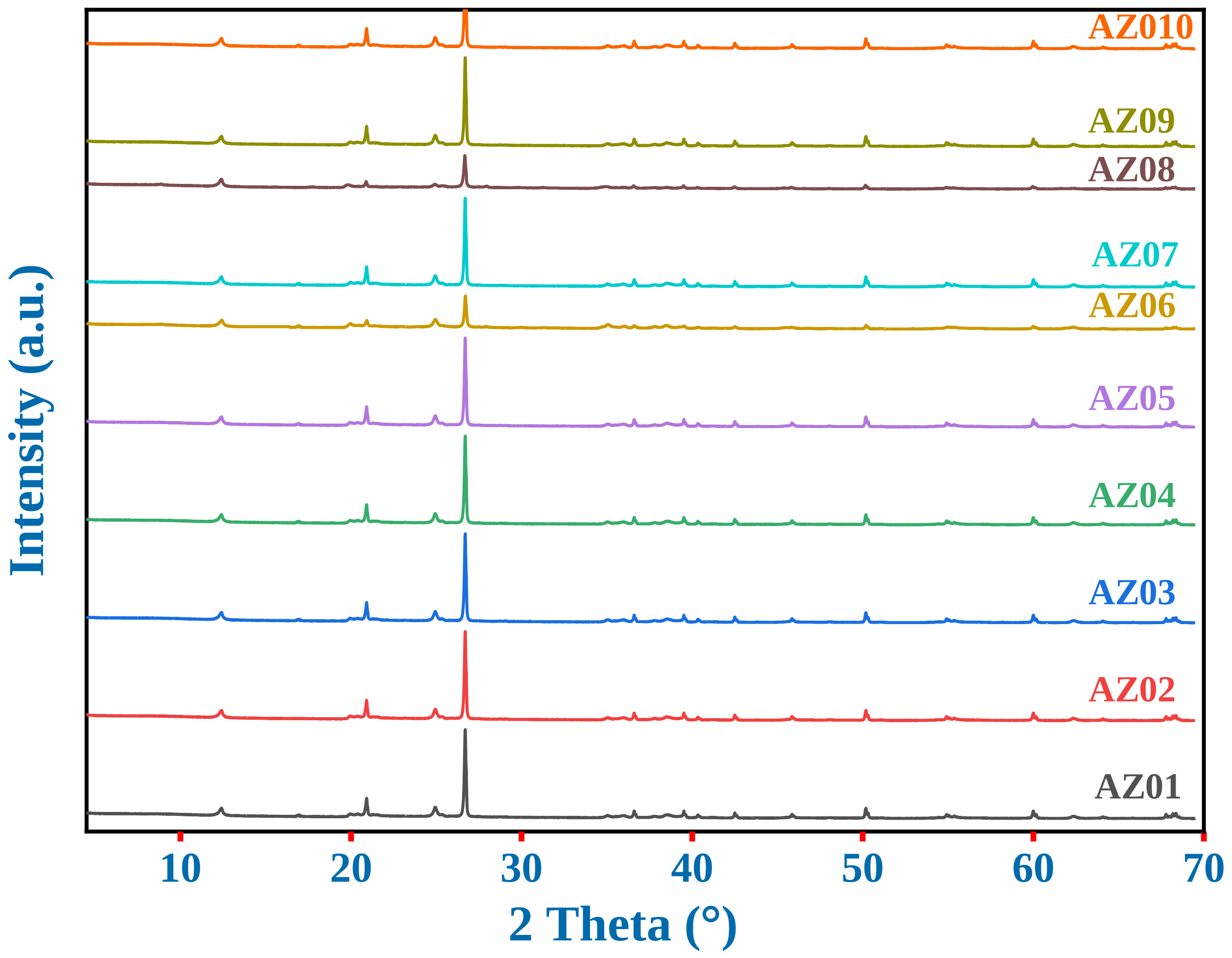

Fig. S2. Shows the XRD patterns of all studied samples.
